# Early individualized positive end-expiratory pressure guided by electrical impedance tomography in acute respiratory distress syndrome: a randomized controlled clinical trial

**DOI:** 10.1186/s13054-021-03645-y

**Published:** 2021-06-30

**Authors:** Huaiwu He, Yi Chi, Yingying Yang, Siyi Yuan, Yun Long, Pengyu Zhao, Inéz Frerichs, Feng Fu, Knut Möller, Zhanqi Zhao

**Affiliations:** 1 Department of Critical Care Medicine, State Key Laboratory of Complex Severe and Rare Diseases, Peking Union Medical College Hospital, Peking Union Medical College, Chinese Academy of Medical Sciences, Beijing, China; 2 Department of Administration, Peking Union Medical College Hospital, Peking Union Medical College, Chinese Academy of Medical Sciences, Beijing, China; 3 Department of Anesthesiology and Intensive Care Medicine, University Medical Center of Schleswig-Holstein Campus Kiel, Kiel, Germany; 4 Department of Biomedical Engineering, Fourth Military Medical University, 169 Changle Xi Rd, Xi’an, China; 5 Institute of Technical Medicine, Furtwangen University, Villingen-Schwenningen, Germany

**Keywords:** Electrical impedance tomography, PEEP titration, ARDS, Organ function

## Abstract

**Background:**

Individualized positive end-expiratory pressure (PEEP) by electrical impedance tomography (EIT) has potential interest in the optimization of ventilation distribution in acute respiratory distress syndrome (ARDS). The aim of the study was to determine whether early individualized titration of PEEP with EIT improved outcomes in patients with ARDS.

**Methods:**

A total of 117 ARDS patients receiving mechanical ventilation were randomly assigned to EIT group (*n* = 61, PEEP adjusted based on ventilation distribution) or control group (*n* = 56, low PEEP/FiO_2_ table). The primary outcome was 28-day mortality. Secondary and exploratory outcomes were ventilator-free days, length of ICU stay, incidence of pneumothorax and barotrauma, and difference in Sequential Organ Failure Assessment (SOFA) score at day 1 (ΔD1-SOFA) and day 2 (ΔD2-SOFA) compared with baseline.

**Measurements and main results:**

There was no statistical difference in the value of PEEP between the EIT group and control group, but the combination of PEEP and FiO_2_ was different between groups. In the control group, a significantly positive correlation was found between the PEEP value and the corresponding FiO_2_ (*r* = 0.47, *p* < 0.00001) since a given matched table was used for PEEP settings. Diverse combinations of PEEP and FiO_2_ were found in the EIT group (*r* = 0.05, *p* = 0.68). There was no significant difference in mortality rate (21% vs. 27%, EIT vs. control, *p* = 0.63), ICU length of stay (13.0 (7.0, 25.0) vs 10.0 (7.0, 14.8), median (25th–75th percentile); *p* = 0.17), and ventilator-free days at day 28 (14.0 (2.0, 23.0) vs 19.0 (0.0, 24.0), *p* = 0.55) between the two groups. The incidence of new barotrauma was zero. Compared with control group, significantly lower ΔD1-SOFA and ΔD2-SOFA were found in the EIT group (*p* < 0.001) in a post hoc comparison. Moreover, the EIT group exhibited a significant decrease of SOFA at day 2 compared with baseline (paired t-test, difference by − 1 (− 3.5, 0), *p* = 0.001). However, the control group did show a similar decrease (difference by 1 (− 2, 2), *p* = 0.131).

**Conclusion:**

Our study showed a 6% absolute decrease in mortality in the EIT group: a statistically non-significant, but clinically non-negligible result. This result along with the showed improvement in organ function might justify further reserach to validate the beneficial effect of individualized EIT-guided PEEP setting on clinical outcomes of patients with ARDS.

*Trial registration*: ClinicalTrials, NCT02361398. Registered 11 February 2015—prospectively registered, https://clinicaltrials.gov/show/NCT02361398.

## Introduction

Positive end-expiratory pressure (PEEP) is often used in acute respiratory distress syndrome (ARDS) with the aim to open collapsed lung regions and keep the lung open. However, inappropriate setting of PEEP may induce further injury to the lung tissue. It remains challenging for the physicians to balance the regional recruitment and overdistension during the PEEP setting. PEEP could be adjusted based on and/or respiratory compliance; however, these global parameters do not accurately reflect the regional lung physiologic responses induced by PEEP [1]. Individualized PEEP setting based on regional respiratory features is gaining great attention.

Electrical impedance tomography (EIT) is a functional imaging tool that can quantify ventilation homogeneity [2, 3], as well as regional alveolar recruitment and overdistension at the bedside [4]. Hence, EIT could provide deep insights into regional ventilation and lung mechanics allowing an individualized PEEP for ARDS patients under mechanical ventilation. More and more clinical studies have validated the use of EIT for guiding the PEEP setting in various clinical conditions such as ARDS, acute hypoxemia, general anesthesia, and postoperative cardiac surgery patients at the bedside [2, 5–13]. Using the ARDS network, low PEEP/FiO_2_ table to set PEEP is easy and popular in the current clinical practice [14]. Since ARDS patients have a highly variable lung recruitability, an individualized PEEP would be desirable. However, to our knowledge, no randomized controlled trial has been conducted to compare the two strategies of setting PEEP using EIT and the lower PEEP/FiO_2_ table in ARDS patients in ICU. Whether an individualized PEEP setting with EIT could improve patient outcomes remains uncertain and needs to be evaluated.

The aim of this randomized controlled study was to explore whether PEEP setting guided by EIT could improve outcomes compared to PEEP/FiO_2_ table from the ARDS network in ARDS patients.

## Materials and methods

This is a single-center, prospective, open-label, randomized controlled trial (ClinicalTrials.gov, NCT02361398). The study was approved by the Institutional Research and Ethics Committee of the Peking Union Medical College Hospital. Informed consent was obtained from all patients or next of kin before data were included into the study.

From November 2018 to September 2020, ICU patients with ARDS were screened for eligibility. The diagnosis of ARDS was based on the Berlin definition [15]. We have included patients with a BMI < 40 suffering from ARDS with PaO_2_/FiO_2_ < 300 mmHg (diagnosed by a senior grade intensivist according to the Berlin definition) who were sedated and mechanically ventilated with an expected duration of controlled mechanical ventilation of more than 24 h and ability to tolerate PEEP titration (up to 21 or 15 cmH_2_O). Patients aged less than 18 years and more than 85 years, pregnant women, and patients at end-stage medical condition were excluded from the study. Contraindications to the use of EIT (automatic implantable cardioverter–defibrillator, chest wounds limiting electrode belt placement, and implantable pumps) were considered.

Moreover, the COVID-19 patients were not included in the present study.

### Randomization

Eligible patients admitted to ICU were enrolled within 24 h and randomly assigned in a 1:1 ratio to the EIT or the control group (PEEP setting by low PEEP/FiO_2_ table). Randomization was achieved with a computer-generated random block design, which was drawn up by an independent operator before the beginning of the study. Treatment allocation was concealed using sequentially numbered, opaque, sealed envelopes. All nurses and other research personnel were blinded to the randomization schedule and block size.

### Intervention

Patients assigned to the control group continued to receive the low-PEEP strategy using the PEEP/FiO_2_ table of the ARDS network protocol [14]. In the EIT group, PEEP titration by EIT was performed at the enrollment. The optimal PEEP determined by EIT was applied for 24 h. Afterward, PEEP was set by the attending physician based on the low PEEP/FiO_2_ table. EIT measurements were taken with PulmoVista 500 (Dräger Medical, Lübeck, Germany). A silicone EIT belt with 16 surface electrodes was placed around the patient’s thorax at the fourth intercostal space level. EIT data were recorded throughout the PEEP titration in the supine position. During this period, all patients were fully sedated using continuous infusion of midazolam, propofol, fentanyl/remifentanil/sufentanil, and/or atracurium to prevent any spontaneous breathing.

**Fig. 1:**
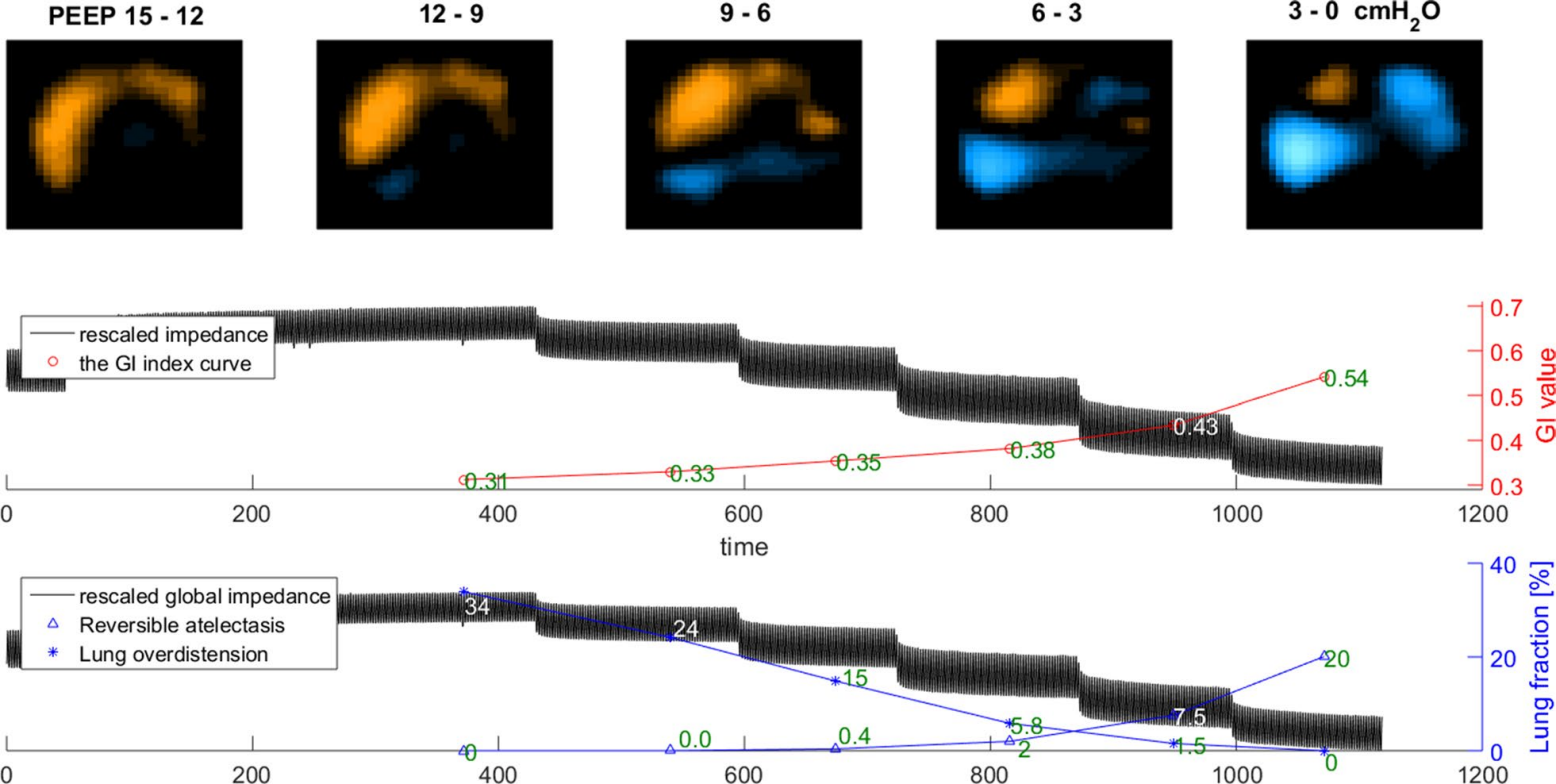
Individualized positive end-expiratory airway pressure titration using electrical impedance tomography in one patient of the EIT group. Optimal PEEP is defined the intercept point of cumulated collapse and overdistension percentage curves, providing the best compromise between collapsed and overdistended lung. If the intercept point occurred between two PEEP steps, the selected PEEP corresponded to the PEEP step toward the lowest global inhomogeneity index. For the presented example, selected PEEP is 6cmH_2_O

The procedure of the EIT-based PEEP titration was as follows: 1. All patients were under pressure control mode (driving pressure 12–15 cmH_2_O with a tidal volume of 6 ml/kg predicted body weight, respiration rate 12–15 bpm). 2. PEEP was increased to 21 cmH_2_O or 15 cmH_2_O for 5 min from baseline. PEEP was increased to 21 cmH_2_O, if the baseline PEEP was higher than 10 cmH_2_O and the patient tolerated the increase, as assessed by the physician (e.g., impaired circulation). Otherwise, PEEP of 15 cmH_2_O was used. 3. PEEP was stepwise decreased from 21 (or 15) cmH_2_O to 0 cmH_2_O in steps of 3 cmH_2_O every 2 min, and FiO_2_ was increased to 1 to maintain oxygenation. If SpO_2_ fell below 88% during the PEEP decrease, the decrease of PEEP was stopped. 4. Optimal PEEP selection: Two EIT-based parameters were calculated. Regional collapse and overdistension percentages were estimated based on the decrease of regional compliance curve calculated during the decremental PEEP trial, toward either lower or higher PEEP levels [16]. The PEEP level selected for the patients in the EIT group was the intercept point of cumulated collapse and overdistension percentage curves, providing the best compromise between collapsed and overdistended lung. If the intercept point occurred between two PEEP steps, the selected PEEP corresponded to the PEEP step toward the lowest global inhomogeneity index [3]. An example individualized PEEP titration by EIT in one patient is shown in Fig. 1. No recruitment maneuver was performed before the PEEP trial. The PEEP value selected according to low-PEEP strategy of the PEEP–FiO_2_ table was noted for individuals in the EIT group, only for comparison purpose.

### Other respiratory and circulatory therapy

Apart from the PEEP selection scheme at day 1, other aspects of care such as small tidal volume ventilation and adjuvant therapies of ARDS were the same for both groups based on local ARDS therapy regulation in our department.

All the patients received local hemodynamic support regimens for critically ill patients. The early goals of hemodynamic support for the tissue hypoperfusion were the following: central venous pressure of 8–12 mmHg; mean arterial pressure above 65 mmHg; urine output above 0.5 ml/kg of body weight (except in the patients with acute renal failure); and central venous O_2_ saturation (ScvO_2_) of 70% or more with the difference between central venous and arterial PCO_2_ (Pv-aCO_2_) of 6 mmHg or less. A negative fluid balance management regimen was used after the correction of shock and/or tissue perfusion.

### Data collection

Patients’ data were collected on an electronic medical platform. The primary endpoint was all-cause mortality within 28 days after randomization. The secondary endpoints included the number of ventilator-free days at day 28 (if a patient died during the 28-day period after enrollment, the number of ventilator-free days was zero), ICU length of stay, new onset barotrauma (pneumothorax, pneumomediastinum, pneumoperitoneum, or subcutaneous emphysema) during mechanical ventilation. The exploratory endpoints were oxygenation and respiratory mechanics, difference in SOFA score at day 1 [17] after randomization minus baseline SOFA score at enrollment (ΔD1-SOFA), as well as the analogous SOFA score difference at day 2 (ΔD2-SOFA).

### Statistical analysis

The sample size was determined to obtain 80% power with an ***a*** level of 0.05 to detect a 25-point difference in 28-day mortality between the two groups (40% in the control group vs 15% in the experimental group) and a sample of 57 in each group. In total, 126 patients were enrolled with the aim to manage the dropouts in this study.

Normally distributed results were presented as mean ± SD, whereas non-normally distributed results were presented as median (25th–75th percentile). Paired data at different time points were compared with the paired sample T test or the Wilcoxon signed rank test. Mann–Whitney test was used to compare groups on continuous variables, and Chi-square and Fisher’s exact tests were used to compare categorical variables. Comparisons of two continuous variables were made using Spearman's correlation. Trend comparisons of the related parameters on different days were performed using a general linear model repeated measures, or so-called repeated measure ANOVA (RM-ANOVA) [18]. This RM-ANOVA model is an extension of the classical ANOVA, which allows handling both fixed effect (different days) and random effect (patient). Bonferroni correction was used to adjust the p value for multiple comparisons. The statistical analysis was performed by using the software package SPSS 24.0 (SPSS Inc., Chicago, IL) and MedCalc 11.4.3.0 Software (Mariakerke, Belgium). A p value smaller than 0.05 was considered statistically significant.

## Results

**Fig. 2:**
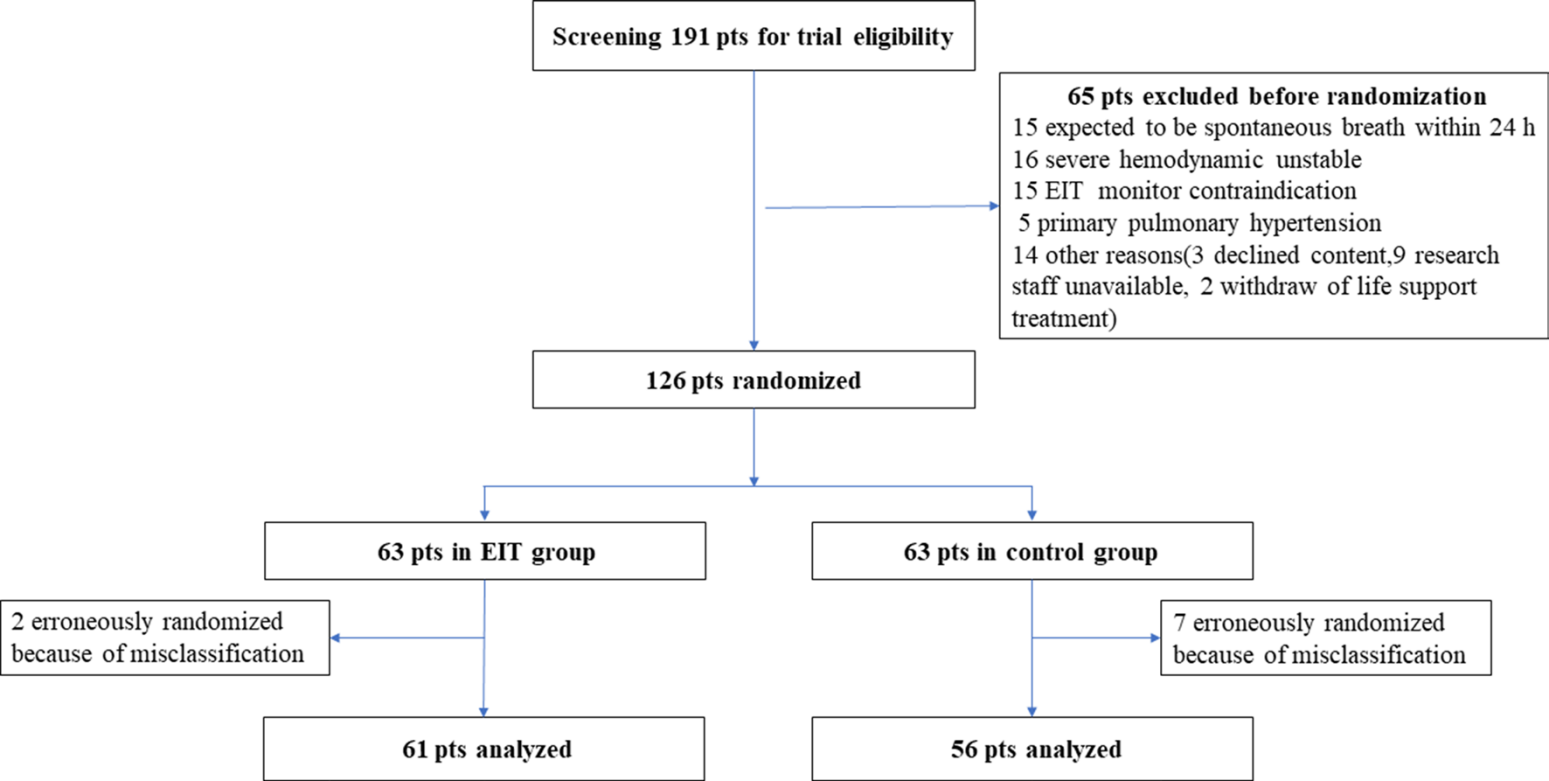
Flowchart of the enrolled patients. pts, patients

A total of 191 ARDS patients were screened and 126 were enrolled: 63 patients in the EIT group and 63 in the control group. Two patients in the EIT group and 7 patients in the control group were erroneously randomized because of misclassification of ARDS (Fig. 2). Thus, 117 subjects (61 EIT group and 56 control group) were included in the primary analysis. No patient was extubated, and 12 patients (four in EIT group and eight in control group) had PaO_2_/FiO_2_ > 300 on the first study day following enrollment.

### Patient characteristics

No significant difference was found in patient characteristics between the two study groups at the baseline (Table 1). Out of 117 patients, 106 received norepinephrine to keep mean arterial pressure (MAP) at the baseline level.

**Table 1:** Baseline clinical characteristics and demographics of patients

Variables	EIT group (*n* = 61)	Control group (*n* = 56)	*p* values
Age (years)	61.0 (44.0, 68.0)	66.5 (50.0, 73.0)	0.074
Female Sex	19/61	21/56	0.597
Body mass index (kg/m^2^)	26.0 (22.9, 29.1)	26.0 (22.9, 28.6)	0.933
APACHE II score	19.0 (15.0, 25.0)	18.0 (15.0, 21.2)	0.568
Reason for ARDS			
Pneumonia	28/61	24/56	0.884
Extrapulmonary sepsis	10/61	15/56	0.252
Severe acute pancreatitis	0/61	1/56	0.972
Post-cardiac operation	14/61	8/56	0.231
Others	9/61	8/56	0.848
Mild ARDS	18/61	23/56	0.292
Moderate ARDS	28/61	25/56	0.960
Severe ARDS	15/61	8/56	0.161
Other parameters			
Heart rate	98.0 (86.0, 113.0)	103.0 (88.2, 120.8)	0.287
Mean arterial pressure	82.0 (73.0, 94.0)	87.5 (75.8, 99.2)	0.242
Received NE (*n*, %)	53/61 (86.9%)	53/56 (94.6%)	0.151
NE dose (ug/kg/min)	0.1 (0.0, 0.3)	0.1 (0.1, 0.2)	0.715
ScvO_2_ (%)	73.7 (71.0, 81.7)	76.2 (68.7, 81.6)	0.953
Pv-a CO_2_ (mmHg)	4.6 (2.6, 6.6)	4.3 (2.4, 7.0)	0.923
Arterial lactate (mmol/L)	2.2 (1.4, 4.7)	2.0 (1.4, 4.0)	0.550
White blood cell (10^^9^/L)	9.8 (8.6, 11.3)	10.3 (8.8, 12.8)	0.287

ARDS, acute respiratory distress syndrome; NE, norepinephrine; ScvO_2_, central venous oxygen saturation; Pv-a CO_2_, venous-to-arterial carbon dioxide difference; APACHE II, Acute Physiology and Chronic Health Evaluation

### Respiratory and arterial blood gas parameters at baseline and on days 0, 1, 2, and 3

Evolutions of related parameters in both the EIT and the control groups at days 0, 1, 2, and 3 are shown in Table 2. There was no difference in the respiratory parameters and arterial blood gas measurements between the groups. Significant and continuous decreases of lactate, SOFA score and APACHE II score, and an increase of pH and PaO_2_/FiO_2_ were found in both groups.

**Table 2:** Difference in respiratory and hemodynamic variables between groups

Parameters	Day 0	Day 1	Day 2	Day 3	Trend *p *value
VT (ml) of PC mode					
EIT group	414 (383, 460)	450 (390, 520) *	430(373, 490)	429 (364, 497)	0.306
Control group	407 (362, 463)	410(370, 445)	411(370, 481)	426 (345, 485)	0.153
Driving pressure (cmH_2_O)					
EIT group	14(11, 15)	13(11, 15)	13(11, 15)	13 (11, 15)	0.631
Control group	13 (11, 15)	13 (12, 14)	12(11.0, 15)	13 (10, 15)	0.772
Pmean (cmH_2_O)					
EIT group	10 (9, 12)	12 (10, 14)	12 (10, 14)	12 (10, 14)	0.065
Control group	10 (9, 12)	11 (10, 13)	12 (9, 13)	11 (10, 13)	0.445
RR (bpm)					
EIT group	15 (15, 15)	15(14, 18)	16(15, 20)	18 (14, 22)	< 0.0001
Control group	15 (15, 16)	16(15, 18)	15(15, 18)	17 (14, 20)	< 0.0001
Respiratory compliance (ml/cmH_2_O)					
EIT group	32 (27, 41)	33 (25, 43)	31 (25, 38)	33 (25, 43)	0.239
Control group	30 (24, 37)	30 (24, 37)	30 (24, 40)	33 (24, 43)	0.150
PEEP (cmH_2_O)					
EIT group	8 (6, 9)	8 (6, 9)	8 (6,9)	8 (6, 9)	0.287
Control group pH	8 (5, 10)	8 (6, 9)	8 (6, 9)	7 (6, 9)	0.249
EIT group	7.4 (7.3, 7.4)	7.4 (7.4, 7.5)	7.4 (7.4, 7.5)	7.4 (7.4, 7.5)	< 0.0001
Control group	7.4 (7.3, 7.4)	7.4 (7.4, 7.5)	7.5 (7.4, 7.5)	7.5 (7.4, 7.5)	< 0.0001
PaCO_2_ (mmHg)					
EIT group	39 (36, 46)	39 (38, 43)	42 (39, 46)	41 (38, 44)	0.239
Control group	40 (35, 43)	41(39, 44)	39 (37, 43)	40 (38, 43)	0.817
PaO_2_ (mmHg)					
EIT group	82 (74, 96)	92 (76, 104)	91 (79, 110)	96 (79, 118)	0.183
Control group	88 (70, 122)	93 (79, 111)	96 (74, 114)	95 (78, 116)	0.984
PaO_2_/FiO_2_ (mmHg)					
EIT group	165 (106, 213)	187 (144, 242)	214 (165, 283)	220 (170, 295)	< 0.0001
Control group	176 (139, 222)	212 (170, 269)	232 (155, 316)	231 (180, 295)	0.001
Lactate (mmol/L)					
EIT group	2.2 (1.4, 4.7)	1.9 (1.2, 2.9)	1.2 (1.0, 2.0)	1.3 (0.9, 1.6)	< 0.0001
Control group	2.0 (1.4, 4.0)	2.0 (1.2, 2.8)	1.6 (1.1, 2.1)	1.4 (1.1, 1.8)	< 0.0001
APACHE II					
EIT group	19 (14,25)	18 (12, 24)	15 (12, 21)	16 (12, 19)	< 0.0001
Control group	17 (14,20)	18 (16, 21)	16 (13, 21)	15 (13, 20)	< 0.0001
SOFA					
EIT group	13 (11, 14)	12 (10, 14)	11 (9, 13)	11 (9,13)	< 0.0001
Control group	12 (9, 13)	12 (11, 14)	12 (10, 14)	12 (10,14)	0.022

*EIT group versus control group, *p* < 0.05. VT, tidal volume; PC, pressure control mode; Pmean, mean airway pressure; RR, respiratory rate; PEEP, positive end-expiratory pressure; APACHE II, Acute Physiology and Chronic Health Evaluation; SOFA, Sequential Organ Failure Assessment

### PEEP selected by EIT and PEEP/FiO_2_ table

There was no statistical difference in the value of PEEP between the EIT group and control group, but the combination of PEEP and FiO_2_ was different between groups. In the control group, a significantly positive correlation was found between the PEEP value and the corresponding FiO_2_ (*r* = 0.471, *p* < 0.00001) in the control group since a given matched table was used for PEEP/FiO_2_ settings. In the EIT group, divergent individual combinations of PEEP and FiO_2_ ranges were found. No correlation was found between the individual PEEP value of EIT titration (PEEP_eit_) and the corresponding FiO_2_ (*r* = 0.053, *p* = 0.684) in the EIT group.

**Fig. 3:**
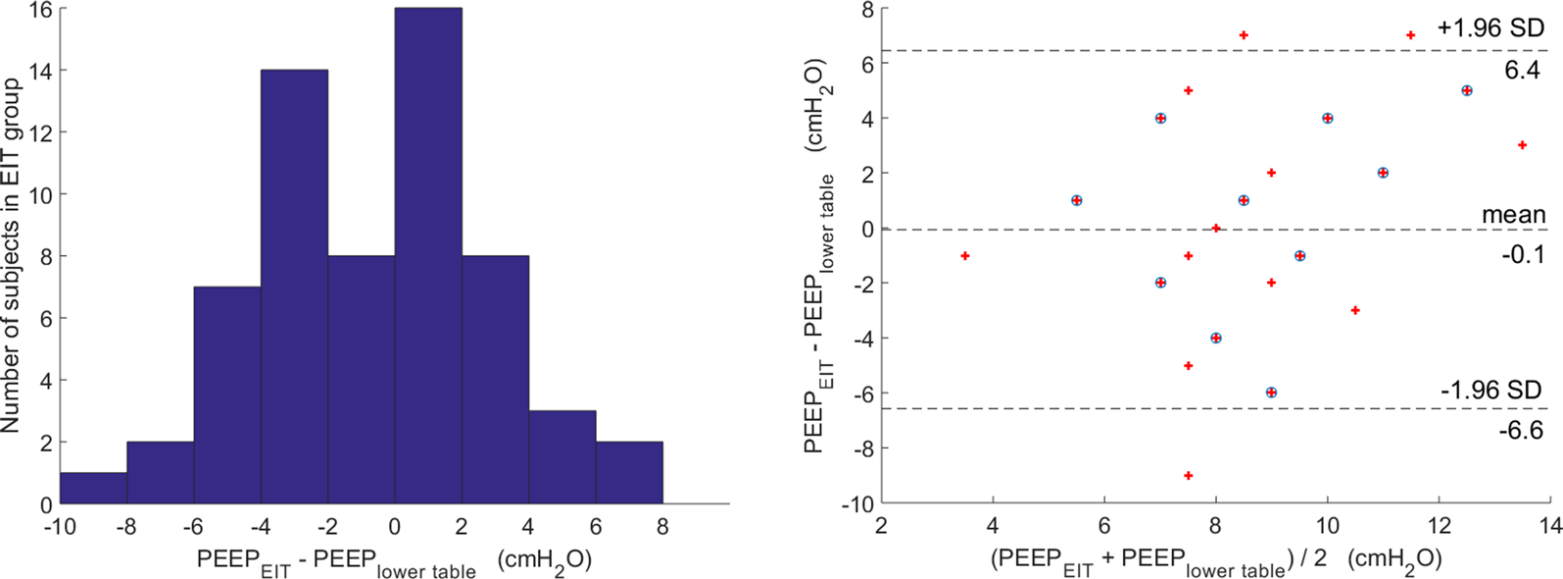
Distribution of the difference values and agreement of Bland–Altman plot between PEEP_eit_ and PEEP_lower table_ methods in the EIT group. Out of 61 patients, 41 exhibited an absolute difference value between PEEP_eit_ and PEEP_lower table_ ≥ 2cmH_2_O. Mean difference between the PEEP_eit_ and PEEP_lower table_ was − 0.1 [95% limits of agreement was from 6.4 to (− 6.6) cmH_2_O]

In the EIT group, 41/61 patients exhibited an absolute difference value between the PEEP_eit_ method and PEEP_lower table_ method ≥ 2cmH_2_O. Distribution of the difference values and agreement of Bland–Altman plot between PEEP_eit_ and PEEP_lower table_ methods are shown in Fig. 3.

### Outcome and adjuvant therapies between groups

**Fig. 4:**
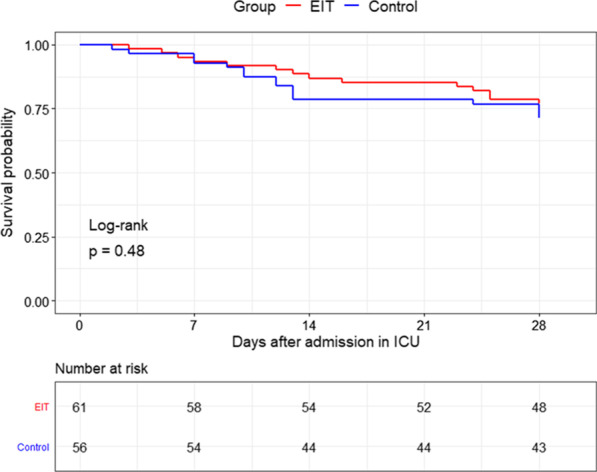
Kaplan–Meier 28-day probability of survival curve for the EIT group and the control group

The outcomes are summarized in Table 3. On day 28 after randomization, the death from any causes had occurred in 13 of 61 patients (21%) in the EIT group and 15 of 56 patients (27%) in the control group (*p* = 0.634) (Table 3 and Fig. 4). There were no significant differences in ventilator-free day at day 28, rate of successful extubations, length of ICU day, and adjuvant therapies of ARDS between the groups (Table 3). The incidence of new barotrauma was zero.

**Table 3:** Main outcome variables and adjuvant therapies in the two study groups

Variables	EIT group *N* = 61	Control group *N* = 56	*p* value
Clinical outcome			
28-day mortality (*n*, %)	13 (21%)	15 (27%)	0. 634
Ventilator-free days at day 28 (D)	14.0 (0.0, 23.0)	18.5 (0.0, 24.0)	0.764
Length of ICU stay (D)	13.0 (7.0, 25.0)	10.0 (7.0, 14.8)	0.169
ΔD1 SOFA score	0 (− 1, 1)	0.5 (− 1, 2.75)	0.021*
ΔD2 SOFA score	− 1 (− 3.5, 0)	1 (− 2, 2)	< 0.0001*
Successful extubation (*n*, %)	30 (49%)	31 (55%)	0.629
Tracheostomy (*n*, %)	17 (28%)	11 (20%)	0.409
Adjuvant therapy			
Neuromuscular blocker (*n*, %)	12 (20%)	5 (9%)	0.166
Prone position (*n*, %)	30 (49%)	23 (41%)	0.487
Glucocorticoid therapy (*n*, %)	11 (18%)	6 (11%)	0.390

*p < 0.05

Significantly lower ΔD1-SOFA and ΔD2-SOFA were found in the EIT group (Table 3). Moreover, the EIT group exhibited a significant decrease of SOFA at day 2 compared with baseline (paired t-test, difference by − 1 (− 3.5, 0), *p* = 0.001). However, the control group did show a similar decrease (difference by 1 (− 2, 2), *p* = 0.131).

Based on different severity of PaO_2_/FiO_2_ at baseline, a subgroup analysis of mortality for the mild–moderate and severe ARDS patients was conducted. For the severe ARDS, 5/15 in the EIT versus 2/8 in the control group died (*p* = 1.00). For the mild–moderate ARDS, 8/46 in the EIT versus 13/48 in the control group died (*p* = 0.26).

## Discussion

In the present study, ARDS patients were randomized and PEEP titration with EIT was compared to low PEEP/FiO_2_ table recommended by the ARDS network. We found that early individual PEEP setting with EIT led to a better but insignificant survival rate. Besides, it might also result in a faster early recovery of organ function.

### Explanations for lack of survival benefits

A randomized controlled clinical trial found that PEEP values determined with EIT effectively improved oxygenation and lung mechanics during one lung ventilation in elderly patients undergoing thoracoscopic surgery [10]. One prospective study with historical control group found the EIT-guided PEEP titration may be associated with improved oxygenation, compliance, driving pressure, and weaning success rate in severe ARDS patients [11]. Recently, compared with the pressure–volume curve method, Hsu et al. found PEEP titration guided with EIT might be associated with improved driving pressure and survival rate in moderate to severe ARDS [19]. In the present study, we did not find statistical significance between the studied groups. There were several potential reasons for lack of survival benefits in the experimental group. First, the patients we enrolled suffered from mild to severe ARDS. In the first working version of the study protocol before its finalization, we planned to include only moderate to severe ARDS (PaO_2_/FiO_2_ < 200 mmHg). With the projected limited number of subjects, we decided to extend the study subjects to mild ARDS as well. The mortality rate was much lower than the one we assumed for sample size calculation and the one from Hsu’s study [19]. Although the trend might be clinically evident (6% difference) given the higher number of severe ARDS in the EIT group (25% vs. 14%), the low mortality rate limited the power of our study to detect a statistical significance between the groups. Second, the design of the study was to explore early PEEP setting guided by EIT and the differences between groups within a short period. One-day intervention time might be too short to validate the impact of optimal PEEP by EIT on the survival and other endpoints (ICU length of stay, length of mechanical ventilation). Repeated regular use of EIT for individualized PEEP setting in the course of the ICU stay might have led to other outcomes. Third, 20/61 patients obtained an individual PEEP value by EIT which was similar to the PEEP setting method of ARDSnet low PEEP/FiO_2_ table. Whether individual PEEP setting by EIT in ARDS can decrease mortality should be assessed in a future larger, possibly multi-center clinical trial.

### Strengths of this study

Reducing morbidity (organ failure) in critically ill patients is intrinsically relevant, and the SOFA score is a valuable endpoint in itself. De Grooth et al. found that ΔSOFA was significantly associated with mortality and explained 32% of the treatment effects on mortality [20]. Since a relatively short intervention period (one day) was applied in this study, ΔD1- and ΔD2-SOFA might be more reasonable endpoints. The organs failure is common at ARDS onset and during the course of ARDS and is associated with mortality [21–23]. Possible explanations for the improvement of organ function recovery in the EIT group are summarized as follows:

1. Individual parameters (lung collapse and overdistension, inhomogeneities) related to lung injury were taken into consideration in the PEEP setting by EIT. In the EIT group, compared to PEEP setting of ARDSnet table, more than 50% (41/61) patients exhibited an absolute difference value between PEEP_eit_ and PEEP_lower table_ ≥ 2cmH_2_O in the EIT group. However, PEEP titration according to the ARDSnet table is less individualized. Hochhausen et al. also found that PEEP setting by EIT facilitates a more individual ventilation therapy in an animal study [24].Wolf et al. confirmed that EIT-guided PEEP selection could improve outcomes in the setting of acute lung injury than the PEEP setting of ARDSnet table in an prospective animal study [24, 25]. Moreover, 5/61 (8%) patients had huge difference (≥ 6cmH_2_O) between PEEP_eit_ and PEEP_table_ methods in the EIT group. The following two conditions were found: 1. EIT suggested that a high PEEP causes significant overdistension but little recruitment during the PEEP titration in some patients with a high FiO_2_. Hence, a low PEEP was determined by PEEP_eit_, whereas a high PEEP was determined by PEEP_table_. 2. EIT suggested that a high PEEP causes a significant regional lung recruitment with little overdistension during the PEEP titration in some patients with a relative low FiO_2_. Hence, a high PEEP was determined by PEEP_eit_, whereas a low PEEP was determined by PEEP_table_. A similar phenomenon of huge difference between PEEP_eit_ and PEEP_table_ was also found in the COVID-19 patients [13]. This result supported the PEEP setting by FiO_2_ might cause a significant lung overdistension or lung collapse in some ARDS patients. Recently, Tsolaki et al. proposed that the PEEP setting based on ARDSnet table might be detrimental in COVID-19 [26].
2. PEEP titration approaches by EIT are based on the assumption that there is an optimal compromise between the limiting the amount of collapse and avoidance of alveolar overdistention. Based on the pathophysiologic theory, the best compromise of regional collapsed and overdistended lung might result in potential benefits regarding circulation and organ functions. Both alveolar collapse and overdistension are harmful during the mechanical ventilation. Moreover, alveolar overdistension exerts a negative effect on the lung circulation and right heart even in the condition of normal oxygenation. Poor right heart function could further impact venous return and then impair the recovery of renal and liver function.
3. No recruitment maneuver was performed before the PEEP trial. A significant improvement in respiratory related parameters (such as oxygenation and respiratory compliance) was not found in the EIT group, and the recovery of organ function might be mainly on the no-respiratory organs. Further studies are required to investigate the effect of PEEP setting by EIT on the individual organs (heart, liver, kidneys, etc.)

### Subset analysis based on ARDS severity

A bigger difference in mortality was observed for the mild–moderate ARDS patients in the subset analysis. The following points should be taken into consideration: 1. The PaO_2_/FiO_2_ might not be accurate reflecting the severity and prognosis in ARDS patients [27, 28]. DesPrez et al. found that the APACHE II but not PaO_2_/FiO_2_ had the greatest performance to predict mortality in ARDS [27]. 2. Mild–moderate ARDSs usually have shorter treatment period compared to the severe ones, and our protocol only involved different PEEP strategies on the first day. 3. The statistical power was limit since the subgroup had insufficient sample size. Moreover, there might be a high risk of the selection bias in the small sample of subset analysis.

### Limitations

Further limitations should be acknowledged. 1.The study was not blinded, and the severities of ARDS in the study groups were different. 2. EIT also had the potential to guide PEEP setting in prone position [29, 30]. Over the entire hospital stay, more than 40% of patients received prone positioning. The effect of prone positioning on the results and outcomes was not analyzed in the present study. 3. The rapidly improving ARDS patients were not excluded in the present study. Twelve of 107 (11.2%) patients were rapidly improving ARDS in our study, which was similar to the previous RCT of ARDS (about 10–15%) [31, 32]. Rapidly improving ARDS might negatively affect the prognostic enrichment and contribute to the failure of therapeutic trials [31]. A considerable within-trial variation in the baseline risk of death was found in the RCT of ARDS [33]. Further study is required to validate the PEEP setting by EIT in the ARDS with less heterogeneity. 4. The primary expected outcome of reduced mortality by 25% was an ambitious target in the initial design of the trial. A recent randomized controlled trial compared PEEP based on EIT and the PV loop in moderate to severe ARDS [19] and reported a ~ 25% reduction in mortality. Nevertheless, by setting such a target, the chance was high that the difference between groups was not statistically significant. The expected mortality would be lower than 40% with a substantial proportion of mild ARDS as in the present study. We acknowledge that the mortality rate of the control group in sample size calculation was only a rough estimation and did not take into account the prevalence of mild ARDS over the years.

## Conclusions

Early individualized PEEP setting by EIT might result in a faster early recovery of organ function. Our study showed a 6% absolute decrease in mortality in the EIT group: a statistically non-significant, but clinically non-negligible result. This result along with the showed improvement in organ function might justify further reserach to validate the beneficial effect of individualized EIT-guided PEEP setting on clinical outcomes of patients with ARDS.

## Key messages

- Early individualized PEEP setting by EIT might results in a faster early recovery of organ function.
- Whether individualized PEEP setting by EIT in ARDS can decrease mortality should be assessed in a future clinical trial.

## Data Availability

The datasets used and/or analyzed during the current study are available from the corresponding author on reasonable request.

## Abbreviations

SOFA: Sequential organ failure assessment
ScvO_2_: Central venous oxygen saturation
Pv-aCO_2_: Venous-to-arterial carbon dioxide difference
APACHE II: Acute Physiology and Chronic Health Evaluation
ARDS: Acute respiratory distress syndrome
GI: Global inhomogeneity index
PEEP: Positive end-expiratory pressures
EIT: Electrical impedance tomography
